# The Science and Art of Surgery

**Published:** 1859-04

**Authors:** 


ART. VII.—*The Science and Art of Surgery. Being a Treatise on Surgical Injuries, Diseases and operations.* By JOHN ERICHSEN, Professor of Surgery and of Clinical Surgery, in University College, and Surgeon to University College Hospital. An improved American edition, from the second enlarged and carefully revised London edition. Illustrated by four hundred and seventeen engravings on wood. Philadelphia: BLANCHARD & LEE, 1859.

We have merely to notice the issue of a new and extended edition of this admirable text book, as it is now favorably known to every student of Surgery. This edition is materially enlarged and has been revised by the author with the greatest care. The volume before us has also received some clippings from the notes of the American editor, in the former edition. We cannot but think, as we remarked above, that a faithful reprint is the best way to present most European books to American readers; there are few points of importance, however, in American Surgery which are not appreciated by our transatlantic bretheren, which it is well enough to introduce into a reprint, but they are not numerous. The new edition of Erichsen's Surgery is brought fully up to the times, and will sustain its reputation as a reliable text book and guide to the practitioner.

